# Characterization and distribution of a 14-Mb chromosomal inversion in native populations of rainbow trout (*Oncorhynchus mykiss*)

**DOI:** 10.1093/g3journal/jkae100

**Published:** 2024-06-17

**Authors:** Matthew C Hale, Devon E Pearse, Matthew A Campbell

**Affiliations:** Department of Biology, Texas Christian University, Fort Worth, TX 76109, USA; Southwest Fisheries Science Center, National Oceanic and Atmospheric Administration, Santa Cruz, CA 95064, USA; Department of Animal Science, University of California Davis, Davis, CA 95616, USA

**Keywords:** chromosomal inversions, homokaryotypes, salmonids, anadromy, structural variation

## Abstract

Multiple studies in a range of taxa have found links between structural variants and the development of ecologically important traits. Such variants are becoming easier to find due, in large part, to the increase in the amount of genome-wide sequence data in nonmodel organisms. The salmonids (salmon, trout, and charr) are a taxonomic group with abundant genome-wide datasets due to their importance in aquaculture, fisheries, and variation in multiple ecologically important life-history traits. Previous research on rainbow trout (*Oncorhynchus mykiss*) has documented a large pericentric (∼55 Mb) chromosomal inversion (CI) on chromosome 5 (Omy05) and a second smaller (∼14 Mb) chromosome inversion on Omy20. While the Omy05 inversion appears to be associated with multiple adaptive traits, the inversion on Omy20 has received far less attention. In this study, we re-analyze RAD-seq and amplicon data from several populations of rainbow trout (*O. mykiss*) to better document the structure and geographic distribution of variation in the Omy20 CI. Moreover, we utilize phylogenomic techniques to characterize both the age- and the protein-coding gene content of the Omy20 CI. We find that the age of the Omy20 inversion dates to the early stages of *O. mykiss* speciation and predates the Omy05 inversion by ∼450,000 years. The 2 CIs differ further in terms of the frequency of the homokaryotypes. While both forms of the Omy05 CI are found across the eastern Pacific, the ancestral version of the Omy20 CI is restricted to the southern portion of the species range in California. Furthermore, the Omy20 inverted haplotype is comparable in genetic diversity to the ancestral form, whereas derived CIs typically show substantially reduced genetic diversity. These data contribute to our understanding of the age and distribution of a large CI in rainbow trout and provide a framework for researchers looking to document CIs in other nonmodel species.

## Introduction

With the advent of next-generation sequencing and high-power computational analyses, it has become easier to generate genetic maps and identify segregating structural variants (SVs), improving their detection (Flagel *et al*. 2019). A lot of interest has recently been generated in identifying and documenting chromosomal inversions (CIs) in natural populations and linking different homokaryotypes (often referred to as “supergenes”) to ecologically important traits. This interest is due to recombination suppression between alternative homokaryotypes through nonviability of recombinant gametes, which results in the protection of locally adapted alleles (Kirkpatrick 2010; Schwander *et al*. 2014; reviewed in Wellenreuther and Bernatchez 2018; Huang and Rieseberg 2020). Although many forms of SVs can suppress recombination, CIs are often large—in the order of several megabases—and therefore are more easily found, described, and documented than smaller SVs such as deletions, insertions, and copy number variants (but see Huang *et al*. 2020; Gao *et al*. 2021 for examples of smaller CIs).

The salmonids (salmon, trout, and charrs) are an exemplary group to study genomics in wild organisms, as many species are economically of interest due to human consumption, and are major recreational fishing, aquaculture and commercial fisheries species. Additionally, numerous salmonids are important to native peoples for cultural and subsistence reasons. These factors have led to near-complete genome sequences, as well as significant resequencing efforts, for several species of salmonid that are crucial resources in understanding variation in genome architecture and the genomic basis to the development of ecologically important traits (Lien *et al.* 2016; Pearse *et al*. 2019; Bertolotti *et al*. 2020). For example, previous investigations have found CIs in chum salmon (*Oncorhynchus keta*) from western Alaska (McKinney *et al*. 2020), in Arctic charr (*Salvelinus alpinus*) from Nunavut, Canada (Hale *et al*. 2021), in Alaskan sockeye salmon (*Oncorhynchus nerka*; Euclide *et al*. 2023), and in Atlantic salmon (*Salmo salar*, e.g. Lehnert *et al* 2019; Stenløkk *et al*. 2022). In rainbow trout (*Oncorhynchus mykiss*), much research has focused on a 55-Mb pericentric inversion on chromosome 5 (Omy05). This inversion contains ∼1,100 protein-coding genes and has been found to be associated with life-history development (i.e. residency vs anadromy) as well as other adaptive traits such as embryonic development rate, sexual maturation, phototransduction, and behavior (Sundin *et al*. 2005; Nichols *et al*. 2007; Hale *et al*. 2014; Pearse *et al*. 2019). However, an additional 14-Mb paracentric inversion on the p-arm of Omy20 with ∼300 protein-coding genes was described by Pearse *et al*. (2019) and Gao *et al*. (2021) in 2 homozygous inbred lines of *O. mykiss*, but has received far less attention. Indeed, only one other publication—to the best of our knowledge—has noted patterns of variation in the Omy20 inversion in wild populations (Campbell *et al*. 2021), and that study was limited to trout from 4 coastal populations of rainbow trout (*O. m. irideus*) located in central to southern California.

The study of Campbell *et al*. (2021) sampled not only a restrictive set of populations, but also a small proportion of rainbow trout phylogenetic diversity. Coastal rainbow trout (*O. m. irideus*) are widely distributed across the North Pacific and represent a more recent postglacial expansion (McCusker *et al*. 2000). Long-term occupancy of California is shown through the diverse lineages present there, such as the McCloud River Trout (*O. m. calisulat*) and the Golden Trout Complex, which may be recognized as a separate species (*Oncorhynchus aguabonita*; Campbell *et al*. 2023). Archaic “redband” trout are present not only as a geographic isolate in the form of McCloud River Trout, but also as more recent dispersers from the Great Basin/Upper Columbia River into California (*O. m. newberrii* and *O. m. stonei*), further increasing the phylogenetic diversity of *O. mykiss* in California (Campbell *et al*. 2023). Given this phenotypic and genetic diversity, the scarcity of data documenting the different forms of the Omy20 inversion makes it difficult to determine the geographic and phylogenetic distribution of the 2 homokaryotypes, the frequency of heterozygotes, or the association between the different forms of the inversion and selected traits. Herein, we analyze preexisting datasets to describe and document the Omy20 CI and to determine the distribution of the 2 karyotypes over a broad geographic range from Alaska to California. In addition, we investigate the roles and functions of the protein-coding genes within the inversion and use sequence information from these genes to estimate the age of the inversion. These results provide information on this less studied CI in rainbow trout, as well as providing an evolutionary framework for estimating the age and recombination patterns within CIs.

## Materials and methods

Two data sources were used to determine the Omy20 karyotype of rainbow trout from the west coast of the contiguous United States and Alaska. First, we re-analyzed previously published reduced representation (RAD-seq) sequencing data downloaded from the GenBank SRA for samples of *O. mykiss* (see Supplementary Table 1 for details of the sampling location, sample size, and the GenBank accession numbers of the raw data). Second, we used data from a newly published amplicon sequencing panel focused on Californian rainbow trout (Le Gall *et al*. 2024; details provided in Supplementary Table 2). These 2 datasets were analyzed separately (see below), and the results were combined to evaluate the inferred frequencies of the Omy20 CI across the sampled populations.

### Analysis of RAD-seq data

RAD-seq data from 701 samples were aligned to Version 2 of the rainbow trout genome (Pearse *et al*. 2019) using bwa-mem v0.7.17 with default parameters (Li and Durbin 2009). SAMtools v1.18 was then used to convert sequence alignments into sorted bam files, and genotypes were determined using ANGSD v0.93 (Korneliussen *et al*. 2014) with the following parameters (snp_pval 1e-6, postCutoff 0.95, minQ 20, minMapQ 20, minMaf 0.05, and minInd 500). The SAMtools model was used for determining genotypes (Li 2011), and the results of ANGSD were outputted in plink format. PLINK1.9 (Purcell *et al*. 2007; Chang *et al*. 2015) was then used to calculate Linkage Disequilibrium (LD) for Omy20 to determine the presence and location of the CI. LD was calculated between all pairs of markers on Omy20 using the following parameters: -ld-window-r2 0, -r2 inter-chr. Estimates of LD were then filtered to include only loci that produced *r*^2^ values >0.8. All samples were then recalled from the existing sequence data using 75 loci that mapped to the CI region of Omy20 and were indicative of the CI karyotypes (i.e. homozygous for the ancestral form, heterozygous, or homozygous for the inverted form). The ancestral karyotype was identified as the inversion orientation that was shared with other *Oncorhynchus* and the inverted homokaryotype as the form of the CI specific to rainbow trout (see Campbell *et al*. 2021, for more details). Heterozygosity was calculated for all loci within the inversion for the 3 karyotypes using PLINK1.9 (Chang *et al*. 2015) with default parameters. The same methods as above were also used to genotype the same samples for the Omy05 inversion in this dataset to compare neutrality statistics and heterozygosity.

### Population genetic analyses of the RAD-seq dataset

Site Frequency Spectrum (SFS) files were generated in ANGSD v0.93 (Korneliussen *et al*. 2014) using datasets for each Omy20 and Omy05 homokaryotype in fish from populations that were variable for the inversion (i.e. those south of the Siletz River). Briefly, 4 bam lists were created for homozygous individuals (1 for the ancestral karyotype and 1 for the inverted karyotype for each CI), and SFS files were created in ANGSD using the following specified parameters (snp_pval 1e-6, minQ 20, minMapQ 20, and minMaf 0.05). The SFS files were then processed to determine both Tajima's *D* and Watterson's theta using a sliding-window analysis with a window size of 50,000 bp and a slide size of 10,000 bp. All windows that were not in the inversion regions and those within the inversions with fewer than 100 segregating sites were removed to increase confidence in the estimates of neutrality statistics. The same statistics were also calculated for rainbow trout from the same populations that were homokaryotypes for the Omy05 inversion and compared with the Omy20 inversion. Significant differences between homokaryotypes for both CIs were determined using a 2-tailed *t*-test assuming unequal variance. All statistics were run in R using an alpha value of 0.05. Heterozygosity was also calculated for each of the homokaryotypes and compared both between genotypes and between CIs. Inversion-wide heterozygosity was calculated in PLINK1.9 by calculating the per sample number of heterozygous genotypes for all polymorphic positions within the CI region. We also estimated Weir and Cockerham's *F*_ST_ for each SNP located along Omy20 between homokaryotypes. These calculations were performed from the SFS files generated in ANGSD, as described above.

### Amplicon sequencing–based Omy20 inversion data

To expand the geographic sampling beyond that in publicly available RAD-seq data, we examined high-throughput amplicon sequencing data available for rainbow trout, focusing on representative California populations (Le Gall *et al*. 2024). The dataset of Le Gall *et al*. (2023) includes a range of ocean accessible coastal rainbow trout and isolated populations from ∼33°N to ∼45°N latitude. We first determined whether any of the 135 amplicons were located within the Omy20 inversion region by using BLASTN optimized for short sequences (blastn-task blastn-short) against Version 2 of the rainbow trout genome. We filtered alignments based on >95% similarity to the rainbow trout genome and occurring on Omy20 within the CI region. Amplicons within this region were then compared with the RAD-seq data (detailed above) to identify alleles associated with each of the 2 homokaryotypes of the CI. These combined genotype data were then imported into R and filtered to geographic sampling locations; frequencies of the inversion types were plotted geographically with latitude on the *x-*axis and the frequency of the ancestral Omy20 CI were plotted with latitude on the *y-*axis.

### Dating of Omy20 and Omy05 inversions

To estimate the age of both Omy20 and Omy05 inversions, we identified 2 individuals from the Pearse *et al*. (2019) dataset from coastal California that were homozygous for alternative forms of the inversions. That is, individual M075219 was homozygous ancestral for both Omy20 and Omy05 inversions, and individual M075289 was homozygous for the derived forms of both Omy20 and Omy05. Sequences of all protein-coding loci from the genomes of both M075219 and M075289 were generated from the genome-wide variants identified by Pearse *et al*. (2019) with vcf2fasta (https://github.com/santiagosnchez/vcf2fasta). Coding regions (CDs) from annotated protein-coding genes were combined to generate 1 FASTA-formatted file for both samples.

Additional sequences for phylogenetic analyses were obtained by downloading protein-coding genes from northern pike (*Esox luciu*s), eastern mudminnow (*Umbra pygmaea*), Atlantic salmon, and European grayling (*Thymallus thymallus*) from SalmoBase (Samy *et al*. 2017). The lake whitefish (*Coregonus clupeaformis*) protein-coding genes were obtained from the NCBI (GCF_020615455.1). For all species, orthologs were determined by using OrthoFinder (Emms and Kelly 2019; program options: -d -t 5 -a 5) and filtered to groups of putative orthologs that are present as single copies in northern pike and eastern mudminnow but as duplicates in all salmonids to increase confidence in ortholog groups. Subsequently, ortholog groups with a gene in the Omy20 or Omy05 CIs were identified for additional analyses.

For each high-confidence ortholog group with a gene in the Omy20 or Omy05 CI, a multiple sequence alignment (MSA) was generated with MAFFT (Katoh *et al*. 2002; Katoh and Toh 2008) and then trimmed with BMGE (Criscuolo and Gribaldo 2010). A maximum-likelihood) gene tree for each of the resulting MSAs was generated with IQTREE with support for nodes assessed with ultrafast bootstrap replicates (Nguyen *et al*. 2015; Hoang *et al*. 2018; -m GTR + G -bb 1000). The topology of each gene tree was evaluated to verify that rooting with northern pike and mudminnow sequences produced a topology with 2 clades of salmonid sequences originating from the salmonid-specific whole-genome duplication, that Atlantic salmon and rainbow trout sequences were most closely related in each salmonid sequence clade, and the inversion-region sequences from rainbow trout exhibited a sister relationship and were not identical.

Ortholog groups meeting these additional topological criteria were analyzed separately with MrBayes 3.2 (Ronquist *et al*. 2012). For each ortholog, a time-calibrated phylogeny was generated by providing a starting tree with esociform sequences as outgroups and the 2 clades of salmonid orthologs constrained with lake whitefish and European grayling as sister taxa and salmonine taxa monophyletic. A relaxed clock was used with 2-state nucleotide substitution model with gamma-distributed rate variation. Three exponentially distributed calibrations were used that are based off fossils, dating the Time to The Most Recent Common Ancestor (TMRCA) of northern pike and eastern mudminnow, offsetexp (85, 110.5; Wilson *et al.* 1992; Campbell *et al*. 2013) and TMRCA of Salmonidae in the 2 homeolog clades, offsetexp (51.8, 67.34; Wilson and Williams 1992; Near *et al*. 2012). The TMRCA of *Salmo* and *Oncorhynchus* in the 2 homeolog clades, offsetexp (27.3, 35.49), is based on dates from molecular studies (Campbell *et al*. 2013; MacQueen and Johnston 2014). The root age of the tree was constrained with a uniform prior from 96.1 to 121.5 MYA based on a divergence time of 112 MYA Esociformes and Salmoniformes (Kumar *et al*. 2022).

Initially, MrBayes analyses of 3 runs with 3 chains each were conducted for increasing run lengths, with convergence assessed after a 25% burn in for a subset of 10 orthologs (sump command in MrBayes). Convergence and sufficient effective sample sizes (≫200) of parameters were reached consistently across orthologs with 50 million generations sampled every 5,000 generations. Phylogenies for all orthologs generated with these parameters and the posterior median of the estimated age of divergence of ancestral and inverted forms of each CI were extracted from the consensus tree files created by MrBayes (sumt command). Following Pearse *et al*. (2019), rainbow trout genes that were invariant were removed for dating CIs. Consensus trees of the Omy20 and Omy05 ortholog time trees were created with the consensus.edges function of the phytools package in R (Revell 2012).

### Functional characterization of the Omy20 inversion

We used a BLAST-based approach to determine the functions of protein-coding genes found within the Omy20 CI. Briefly, protein-coding sequences for all 311 genes (as described in Pearse *et al*. 2019) were downloaded from Version 2 of the rainbow trout genome and annotated against the zebrafish reference protein database using BLASTX with default parameters (apart from: maximum *e*-value = 1.0e−10, maximum number of blast hits saved per sequence = 15). BLAST hits were then uploaded into Blast2GO v6 (Conesa *et al*. 2005) to obtain Gene Ontology (GO) terms associated with the zebrafish protein sequences. A Fisher's exact test was used to test for enrichment of protein-coding genes within the Omy20 CI by comparing GO terms associated with the 311 genes within the Omy20 CI and the rest of the protein-coding genes within the rainbow trout genome. Significantly enriched GO terms were identified using a Benjamini–Hochberg False Discovery Rate (FDR)-corrected *P*-value (alpha = 0.05).

## Results

### Geographic distribution of the Omy20 CI karyotypes

**Fig. 1.**
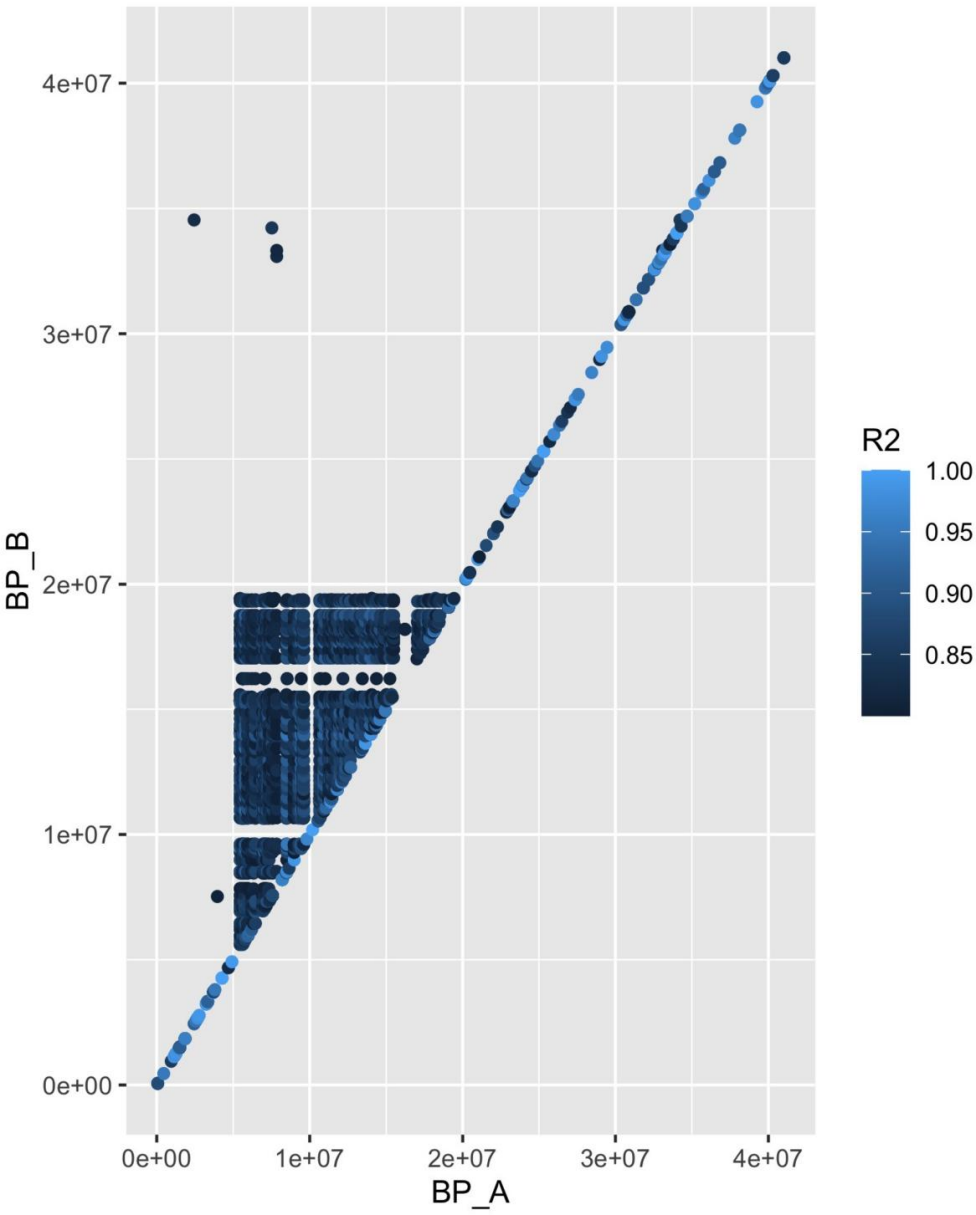
Patterns of LD measured between pairs of loci on Omy20. Only estimates of LD ≥*r*^2^ values of 0.8 are shown for simplicity. The location of the CI is determined to be between 5.6 and 19.2 Mb.

**Fig. 2.**
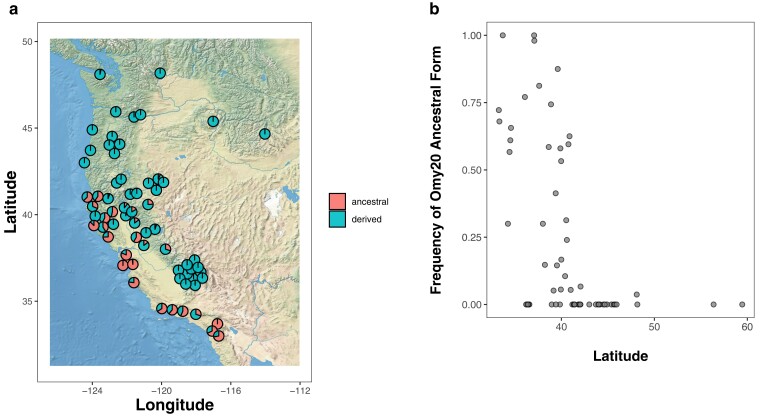
Geographic distribution of Omy20 inversion types within Rainbow Trout from 69 populations (21 from RAD-seq data, 31 from amplicon data, and 17 from both datasets). Sample location, sample sizes, and frequencies of inversion types are reported in Supplementary Tables 1 and 2. Note that hatchery populations and populations with unclear ancestry are not shown. The frequency of ancestral and inverted types is plotted geographically in (a) with locations north of 50° latitude (*n* = 2) omitted, and (b) latitude on the *x-*axis is plotted vs frequency of the ancestral form the *y-*axis.

LD analysis of 421 RAD-seq SNPs that mapped to Omy20 identified 75 SNPs that are indicative of the Omy20 CI karyotype (Pearse *et al*. 2019; Campbell *et al*. 2021). The Omy20 CI spans 13.7 Mb and is located between 5.6 and 19.2 Mb in the rainbow trout genome (Fig. 1). Our RAD-seq data analyses of 41 populations (Supplementary Table 1) revealed a large difference in the frequency of the homokaryotypes with 591 out of 701 samples (84.3%) being homozygous for the inverted form of the inversion, whereas 66 (9.4%) individuals were heterozygous, and 44 (6.3%) were homozygous for the ancestral karyotype. Homozygotes for the ancestral form were restricted to California, with the karyotype being fixed in Scott Creek, and at moderate-to-high frequency in Matilija Creek, Big Creek, the San Gabriel River, and the Eel River (63.3, 86.8, 30.0, and 58.0%, respectively; Fig. 2). Examination of the amplicon sequencing dataset of Le Gall *et al*. (2024) identified 1 microhaplotype locus (Omy_1521) that mapped within the Omy20 inversion region, with alternate alleles that are diagnostic for the ancestral and inverted karyotypes. Population frequencies of the Omy20 ancestral form from Le Gall *et al*. (2024) were estimated based on this locus and merged with the Omy20 haplotype frequencies inferred from the RAD-seq data. In the combined dataset, the ancestral form Omy20 CI form is abundant from ∼33°N to ∼41°N, but rapidly drops in frequency, being absent at ∼44°N in anadromous populations except for the Elwha River, Washington (Fig. 2; Supplementary Tables 1 and 2), and no ancestral homozygotes were found in any population outside of California. There were populations outside of California with the heterozygous karyotype, but these were rare compared with the frequency of the inverted homokaryotype (e.g. 3.7% in the Elwha River, Washington). Interestingly, we did find evidence for heterokaryotypes in 4 redband trout (*O. m. stonei*, Pit River and Surprise Valley regions, California), but note that this was a small percentage of the total number of redbands analyzed (1.4%; data not shown), suggesting the ancestral form is rare and is likely due to the movement of hatchery fish (that were presumably ancestral for the Omy20 CI) and subsequent hybridization with redband trout.

### Population genetics statistics of the homokaryotypes

**Fig. 3.**
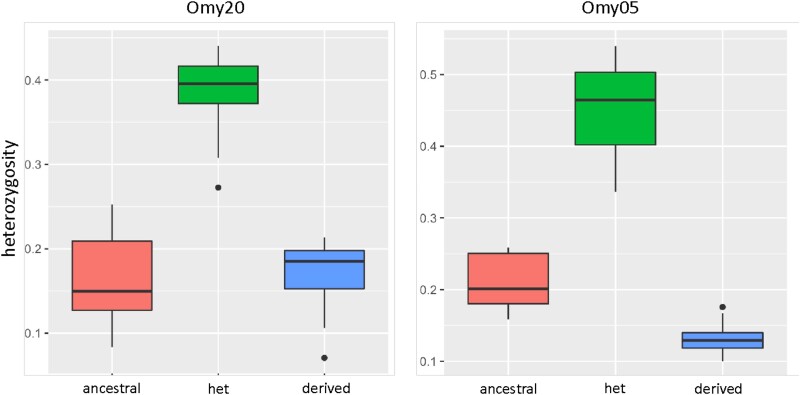
Comparisons of heterozygosity for 76 fish from southern California. Each sample was genotyped at both the Omy20 and Omy05 CIs using 75 and 94 SNPs within these inversion regions, respectively. Heterozygosity was calculated in PLINK1.9. Differences in CI wide heterozygosity were tested using ANOVA: the differences between homokaryotypes were statistically significant for the Omy05 inversion and not for the Omy20 inversion.

**Fig. 4.**
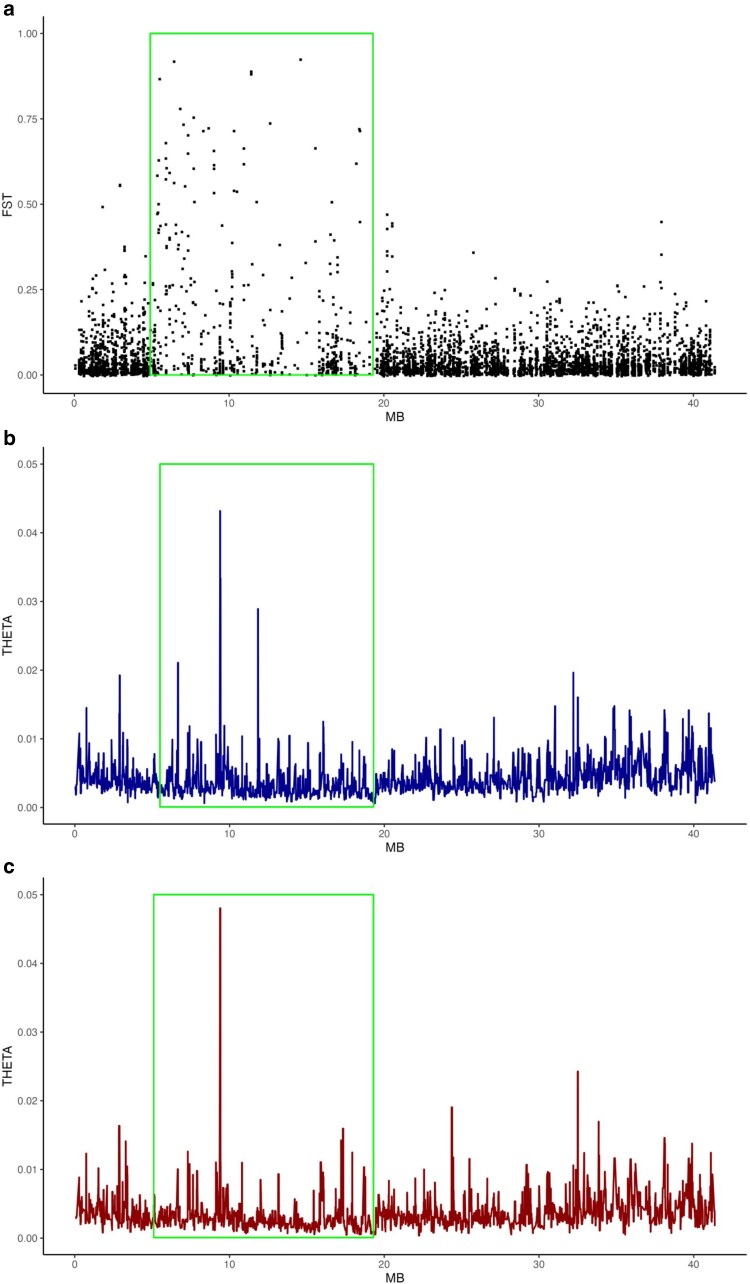
Divergence among Omy20 CI homokaryotypes in *O. mykiss* from southern California. a) Nucleotide divergence (pointwise *F*_ST_) between alternative homokaryotypes reveals the Omy20 CI as an area of elevated divergence. b) and c) show sequence diversity (*π*) among the ancestral and derived homokaryotypes. Estimates of sequence diversity are based on 50 kb windows. The location of the CI is indicated by a box.

Differences in heterozygosity between the homokaryotypes are reduced for the Omy20 CI compared with the different forms of the Omy05 homokaryotypes (not significant between Omy20 homokaryotypes: *t* = 0.105, *P* = 0.456; significant between Omy05 homokaryotypes, *t* = 7.427, *P* ≤ 0.001; Fig. 3). These results imply that the Omy20 inversion is older than the Omy05 inversion, as the inverted homokaryotype has had more time to accrue mutations than the inverted Omy05 homokaryotype, although the combined effects of drift and selection make it difficult to determine based on patterns of sequence variation (Stenløkk *et al*. 2022). We further investigated this difference in age between the 2 CIs by calculating *F*_ST_, Watterson's theta, and Tajima's *D* for the homokaryotic individuals for Omy05 and Omy20. Our calculations of pairwise *F*_ST_ show the location of the Omy20 CI, with multiple loci producing high *F*_ST_ values compared with regions outside the CI (Fig. 4a). The Omy20 homokaryotypes showed similar patterns of Watterson's theta (*t* = 2.234, *P* = 0.013), with the ancestral homokaryotype showing marginally higher estimates of theta than the inverted form (although note that there do not appear to be differences in the distribution of theta values between the CI and non-CI regions of Omy20 (Fig. 4b and c). In contrast, the Omy05 homokaryotypes showed much greater differences in theta values between the different forms of the CI (*t* = 12.303, *P* < 0.001; data not given), suggesting the Omy05 inversion is more recent than the Omy20 CI. Tajima's *D* results were significant for both tests, with the inverted homokaryotype of the Omy05 CI showing significantly negative values compared with the ancestral (*t* = 4.576, *P* < 0.001; data not shown), suggesting the inverted form of the CI is accruing rare polymorphisms.

### Dating of Omy20 and Omy05 inversions

**Fig. 5.**
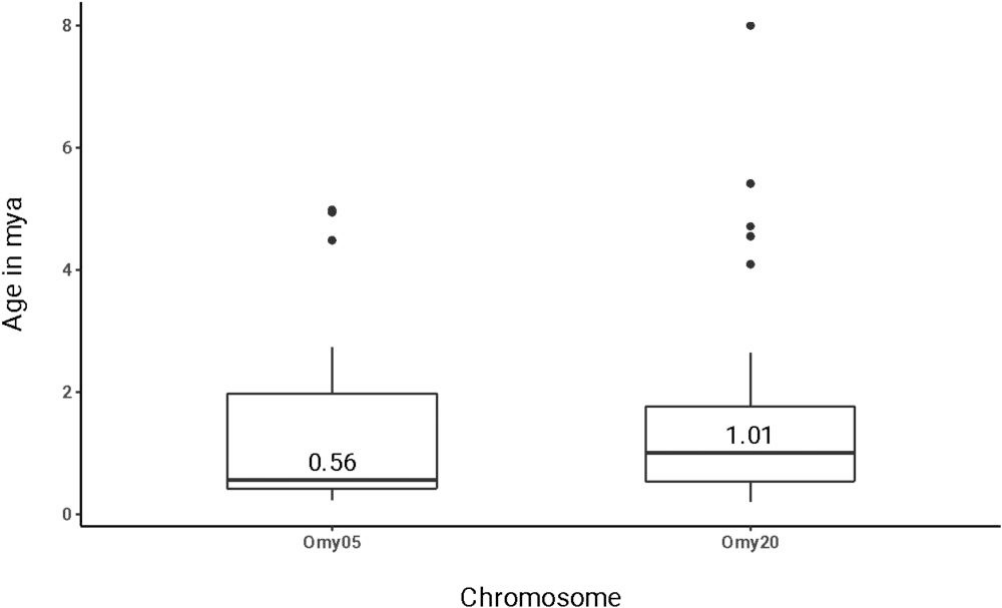
Age of large inversions in rainbow trout. The median age of divergence times is indicated by the orthologs found across each inversion. These estimates are from the analysis of 38 protein-coding genes from the Omy20 CI and 44 genes from the Omy05 CI.

The initial ortholog search identified a set of 34,294 genome-wide orthologs, of which 3,387 were single-copy orthologs in pike outgroups but duplicated in salmonids. Of these orthologs, 55 were located within the CI on Omy20, of which 44 met our requirements for topology (2 ohnolog clades present, sister relationships between rainbow trout and Atlantic salmon in each clade, inversion gene sequences from ancestral and derived forms most closely related). After requiring variation in rainbow trout genes to be present, 38 protein-coding genes were kept for estimating the age of the Omy20 CI inversion. Similarly, for the Omy05 inversion, a set of 266 high-confidence orthologs located within the Omy05 inversion was reduced to an inversion-wide set of 50 genes, with final filtering reducing the total number to 44 genes after orthologs with invariant rainbow trout genes were removed. The older age of the Omy20 CI compared with the Omy05 CI was supported by phylogenetic analyses (Fig. 5, Supplementary Fig. 1). The median age estimated for divergence of the Omy20 CI is ∼1.00 MYA, with the median age of the Omy05 inversion of 0.56 MYA. The mean estimated ages are 1.61 and 1.20 MYA, respectively.

### Functional characterization of the Omy20 inversion

Enrichment tests found 128 overrepresented GO terms in the protein-coding genes located within the Omy20 CI compared with the rest of the genome. Of these terms, 8 were associated with Cellular Component, 25 were with Molecular Functions, and 97 were with Biological Processes. Of the Biological Processes, 41 terms produced FDR-corrected *P*-values <0.00001 and are reported in Table 1. Although making inferences regarding the biological importance of enriched GO terms is difficult, it is striking that many GO terms connected to Wnt-signaling pathways, general development, and ion regulation were found to be over-present in the Omy20 CI. The Omy05 CI contains multiple genes connected to development, phototransduction, photoperiod recognition, and anadromy.

**Table 1.** List of enriched GO terms within the Omy20 CI with a maximum FDR-corrected *P*-value of 0.00001.

GO ID	GO term	*P*-value	FDR *P*-value
0010456	Cell proliferation in dorsal spinal cord	1.59E−08	4.99E−12
0010226	Response to lithium ion	1.59E−08	5.82E−12
0060847	Endothelial cell fate specification	2.39E−08	1.05E−11
0060827	Regulation of canonical Wnt-signaling pathway involved in neural plate anterior/posterior pattern formation	5.94E−08	3.04E−11
0060829	Negative regulation of canonical Wnt-signaling pathway involved in neural plate anterior/posterior pattern formation	6.54E−08	3.86E−11
0060823	Canonical Wnt-signaling pathway involved in neural plate anterior/posterior pattern formation	6.54E−08	4.30E−11
0060784	Regulation of cell proliferation involved in tissue homeostasis	7.49E−08	5.48E−11
0048873	Homeostasis of number of cells within a tissue	7.63E−08	6.14E−11
0007004	Telomere maintenance via telomerase	8.87E−08	9.08E−11
0006278	RNA-templated DNA biosynthetic process	8.87E−08	9.08E−11
0060729	Intestinal epithelial structure maintenance	1.82E−07	2.13E−10
0010833	Telomere maintenance via telomere lengthening	2.24E−07	2.94E−10
0030277	Maintenance of gastrointestinal epithelium	3.14E−07	4.42E−10
0035545	Determination of left/right asymmetry in nervous system	3.14E−07	4.83E−10
0035462	Determination of left/right asymmetry in diencephalon	3.14E−07	4.83E−10
2000054	Negative regulation of Wnt-signaling pathway involved in dorsal/ventral axis specification	3.55E−07	5.71E−10
0019747	Regulation of isoprenoid metabolic process	4.52E−07	8.59E−10
1900052	Regulation of retinoic acid biosynthetic process	4.52E−07	8.59E−10
0032350	Regulation of hormone metabolic process	4.52E−07	8.59E−10
0030656	Regulation of vitamin metabolic process	5.09E−07	1.01E−09
0045995	Regulation of embryonic development	1.12E−06	2.30E−09
0002138	Retinoic acid biosynthetic process	1.14E−06	2.43E−09
0021979	Hypothalamus cell differentiation	1.16E−06	2.55E−09
0010669	Epithelial structure maintenance	1.19E−06	2.78E−09
0016102	Diterpenoid biosynthetic process	1.95E−06	4.70E−09
0042180	Cellular ketone metabolic process	2.35E−06	5.84E−09
0016114	Terpenoid biosynthetic process	3.09E−06	8.14E−09
0042573	Retinoic acid metabolic process	3.58E−06	9.69E−09
0050769	Positive regulation of neurogenesis	3.58E−06	9.94E−09
0050821	Protein stabilization	4.64E−06	1.32E−08
0044283	Small molecule biosynthetic process	5.39E−06	1.58E−08
0072148	Epithelial cell fate commitment	6.20E−06	1.86E−08
0021879	Forebrain neuron differentiation	6.67E−06	2.05E−08
0021872	Forebrain generation of neurons	7.36E−06	2.41E−08
0060839	Endothelial cell fate commitment	7.36E−06	2.42E−08
2000053	Regulation of Wnt-signaling pathway involved in dorsal/ventral axis specification	7.59E−06	2.68E−08
0000723	Telomere maintenance	7.59E−06	2.72E−08
0032200	Telomere organization	7.96E−06	2.91E−08
0072330	Monocarboxylic acid biosynthetic process	9.35E−06	3.49E−08
0060323	Head morphogenesis	9.45E−06	3.59E−08
0021513	Spinal cord dorsal/ventral patterning	9.54E−06	3.77E−08
0010456	Cell proliferation in dorsal spinal cord	1.59E−08	4.99E−12

Each term was enriched for protein-coding genes within the CI compared with protein-coding genes outside the CI.

## Discussion

The rarity and limited geographic range of the ancestral inversion suggest that selection for the inverted form of the Omy20 CI caused an increase in frequency during the colonization of the Pacific Northwest by rainbow trout. Presumably, the restriction of the ancestral version to (mostly) anadromous populations of coastal rainbow trout (*O. m. irideus*) in the southern portion of the species’ range is because it encodes benefits to anadromous migrations or to local climate conditions, and that these benefits are lost in both land-locked resident populations and higher latitude anadromous populations. Pearse *et al*. (2019) proposed sex-dependent balancing selection as a mechanism behind the maintenance of both forms of the Omy05 CI in rainbow trout (anadromous individuals benefit from the ancestral form and resident individuals from the inverted form), and it is tempting to suggest that the 2 forms of the Omy20 CI may also be maintained by some form of balancing selection. An alternative hypothesis is that dispersal and colonization of new environments after the last ice age were predominated by fish with the inverted version of the Omy20 CI, because those individuals were predisposed to dispersal, had a selective advantage, or simply because of the random effects of drift. These hypotheses are not mutually exclusive, especially when one considers that many anadromous steelhead in northern latitudes are homozygous for the inverted forms of both Omy05 and Omy20 CIs (e.g. Hale *et al.* 2013; Weinstein *et al.* 2019), demonstrating that anadromy can and does occur in fish without the ancestral form of either CI.

Given the association (albeit more restricted in geographic range) between the 2 Omy20 homokaryotypes and anadromy, it is tempting to suggest that selection for different developmental processes has maintained the 2 homokaryotypes—at least in California (Campbell *et al*. 2021). However, it is important to note that anadromy is a widespread behavior for rainbow trout across the species’ range, and there was no indication of the ancestral homokaryotype in steelhead from Oregon (e.g. Little Sheep Creek, Siletz, Hood, Willamette, and Umpqua Rivers), Washington (the Elwha, Methow, Klickitat, and Lewis Rivers), and Alaska (Sashin Creek and potadromy in Lake Iliamna). Ultimately, although many of the enriched GO terms are associated with development, the functions of the terms suggest that many of the protein-coding genes within the Omy20 CI are connected to different pathways. This is not surprising, as the large size of the CI encompasses 311 protein-coding genes. Further research into how the 2 homokaryotypes are connected to different phenotypes is necessary to more fully understand how selection has maintained the 2 forms of the CI.

Ultimately, the characterization of these alternative forms of the Omy20 CI, together with our population genetic analyses, increases the knowledge of this so far understudied CI in rainbow trout. Although Campbell *et al*. (2021) suggested a link between the Omy20 CI and migratory life history, we recommend caution for 2 reasons: (1) The ancestral form of the CI is nearly absent north of 42°, at least in the populations sampled (reemphasizing the idea that anadromy and life-history development are controlled by both population-level-specific genetic mechanisms and the environment, Kendall *et al.* 2015), and (2) the lack of phenotypic data associated with the investigated samples makes it impossible to associate the effects of the 2 homokaryotypes on specific traits. This finding contrasts with many studies that note the association of the Omy05 CI and migratory life history in multiple populations of anadromous rainbow trout throughout the Pacific (e.g. Pearse *et al*. 2014, 2019; Arostegui *et al*. 2019; Fraik *et al.* 2021), as well as other related life-history traits (development rate and juvenile growth rate), despite a strong cline in the frequency of the inverted haplotype (Pearse *et al*. 2019). What is clear from our analyses is that the Omy20 CI predates the Omy05 CI, suggesting that the derived form of the CI increased in frequency after expanding from southern glacial refugia into the Pacific Northwest. Moreover, the lack of evidence for the ancestral form of the CI in interior forms of rainbow trout strongly points to the inverted form of the CI increasing in frequency during colonization.

Identification of CIs often relies on comparing the genetic diversity of homozygous and heterozygous individuals, and ancestral heterozygosity is expected to exceed inverted heterozygosity (e.g. Hale *et al*. 2021). While this pattern is true for the Omy05 CI in rainbow trout, it is not true for the Omy20 CI (Fig. 3). We find that the age of the Omy20 CI exceeds that of the Omy05 CI and that the ancestral Omy20 CI is geographically and phylogenetically restricted. In the case of the Omy20 CI, the age of the inversion has substantial impacts on the observed levels of diversity, a factor that should be considered by others as more and more studies document and characterize the presence of CIs in nonmodel organisms (Wellenreuther and Bernatchez 2018).

## Supplementary Material

jkae100_Supplementary_Data

## Data Availability

All sequence data analyzed in this study are publicly available with repository information available in Supplementary Tables 1 and 2. Supplemental material available at G3 online.
