# Supplementary material for: Characterization and distribution of a 14-Mb chromosomal inversion in native populations of rainbow trout (Oncorhynchus mykiss)
Source: G3 (Bethesda). 2024 Jun 17;14(7):jkae100. doi: 10.1093/g3journal/jkae100 (PMC11228831; doi:10.1093/g3journal/jkae100)
Supplement: jkae100_Supplementary_Data [file jkae100_supplementary_data.zip › Supplemental_Figure_1_G3-2024-405001.docx]

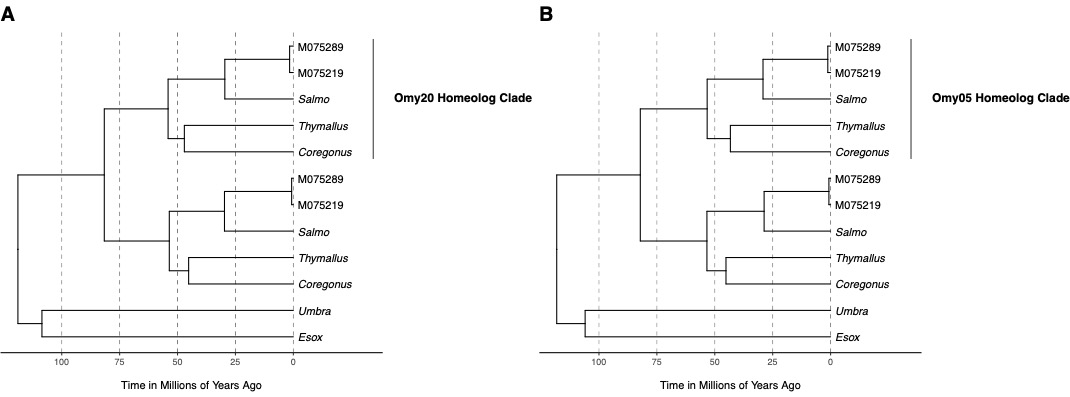


**Supplemental Figure 1** Time-calibrated consensus trees dating chromosomal inversions (A) omy20, n = 38 gene trees (B) omy05, n=44 gene trees. The clades containing inversion homeologs are indicated.
